# Diagnostic Dilemma and Management Difficulties in a Young Patient With Psychosis and Benign Chorea: A Case Report and Review of the Literature

**DOI:** 10.7759/cureus.28177

**Published:** 2022-08-19

**Authors:** Joyce Meza-Venegas, Neil S Kidambi, Alyne Rodrigues, Susan D Sperry, James L Megna, Luba Leontieva

**Affiliations:** 1 Psychiatry, State University of New York Upstate Medical University, Syracuse, USA; 2 Psychology, State University of New York Upstate Medical University, Syracuse, USA; 3 Psychiatry and Behavioral Sciences, State University of New York Upstate Medical University, Syracuse, USA

**Keywords:** anxiety, depression, bipolar disorder, ptsd, schizophrenia, psychosis

## Abstract

Psychosis presents with hallucinations, delusions, disorganized speech, abnormal psychomotor behavior, and negative symptoms. It most commonly appears in the setting of schizophrenia, although it could also appear in bipolar disorder, major depression, post-traumatic stress disorder (PTSD) and even in medical conditions and substance use. In young people, the diagnosis of psychosis can present as a challenge due to the overlap of psychotic conditions and other emotional, behavioral, and developmental disorders. In this case report, we present the case of a 19-year-old female with a history of bipolar disorder, oppositional defiant disorder (ODD), depression, anxiety, PTSD, and schizophrenia-spectrum disorder who was admitted to an inpatient psychiatric facility after presenting with acute onset of confusion.

## Introduction

Psychosis is a clinical syndrome composed of several symptoms which occur in a wide range of mental disorders and show a high degree of interindividual variability between persons with different mental disorders, and a high degree of intraindividual variability over time [1]. The diagnostic criteria for schizophrenia and other psychotic disorders refer to five domains of psychopathology: hallucinations, delusions, disorganized speech, abnormal psychomotor behavior, and negative symptoms [2]. Symptoms of psychosis are usually embedded in the wider clinical picture of the mental disorder, which may include symptoms of mania and depression [1]. The diagnosis of psychotic disorders in children and adolescents can present as complex and challenging and the high rate of misdiagnosis is mostly attributed to the symptomatic overlap between different psychotic conditions and other emotional, behavioral, and developmental disorders [3]. In many cases, young people may be described as having childhood schizophrenia, yet they do not display sufficient psychotic symptoms to meet criteria for the disorder [4]. Histories of abuse and neglect are commonly found in young patients with severe mental health illnesses, including psychotic disorders. One of the many challenges in these cases is to sort abuse-related posttraumatic phenomena from true psychosis [5]. Clinical assessment in psychosis depends as much on input from parents and teachers as from the patients themselves, and there may be conflict between these different perceptions [4]. Here we report a case of a young patient with psychotic symptoms in the setting of multiple psychiatric diagnoses, substance use, several trauma experiences and neglect and a hereditary neurological illness. 

## Case presentation

A 19-year-old female with previous psychiatric diagnoses of bipolar disorder, oppositional defiant disorder (ODD), depression, anxiety, post-traumatic stress disorder (PTSD), and schizophrenia was brought in by Emergency Medical Services (EMS) for evaluation of an acute onset of confusion. The patient was reportedly experiencing auditory hallucinations and confusion and was making nonsensical statements while at a bus stop for which EMS was called. In the ambulance, the patient refused to answer questions. On arrival to the hospital, she appeared confused and stated “I think I’m 19 years old” when asked for her age. She endorsed auditory hallucinations of voices telling her that her mother was dead. She mentioned the voices had become more persistent lately and would sometimes tell her to hurt other people. The patient exhibited a significantly depressed mood, was tearful and did not eat when meals were brought to her. She was noted to have significant thought blocking, delayed thought process during initial conversation, slowed and sometimes muffled speech, flat affect, and delayed cognition. She repeatedly reported that she wanted to go home although she was not able to recall the address. Even though the patient did not endorse suicidal or homicidal ideation, during her stay she had an incident where she put her sheet around the neck and attempted to self-strangulate after which she stated she “just wanted to fly.” 

Family history

The patient’s biological mother reportedly had mental health diagnoses of bipolar disorder, factitious disorder imposed on another, alcohol use, and unspecified personality disorder. The patient’s biological father had a medical diagnosis of autosomal dominant choreoathetosis, a movement disorder that causes involuntary movements of the limbs, trunk, or facial muscles. Her biological sister reportedly had intellectual disability, major depressive disorder, and autosomal dominant choreoathetosis. 

Personal history

Per chart review, the patient had a history of sexual and physical abuse, and neglect by her biological parents which led to her adoption at age 6, along with her other biological sister. During her middle and late childhood, the patient attempted suicide via suffocation and jumping off a second story window at school when she was 13 years old. Between ages 13 and 18, she stayed in several residential treatment facilities where she would become violent with the staff, break windows, and try to escape. During this time, she began using marijuana at an unknown frequency, and engaged in self-injurious behavior via cutting. The patient claimed to hear voices of people who would talk about her in a negative way, for which she “wanted to slit their throats.” Also, she reportedly had frequent delusions about being a witch and having a third eye. The patient somehow managed to make significant progress in school and graduated from high school at age 18 from with a Regents diploma. She had an individual education plan (IEP) in school for “emotional disturbance” due to her multitude of internalizing and externalizing behaviors. The patient had ongoing substance abuse with marijuana and amphetamines and had a history of violent behavior towards her biological and adoptive siblings. She repeatedly threatened her family multiple times with knives and box cutters and verbally stated that if she had access to a gun she would “go through it.” For these reasons, her adoptive mother decided to expel the patient from the house, although she continued to be in communication with her. At the time of admission, the patient was homeless and had been “couch surfing” in friends’ houses during the prior year. The patient had had intense, conflict-filled relationships. Her last partner would allegedly drug her and make her work as a prostitute. 

Psychiatric diagnoses and pharmacotherapy

In adolescence, this patient was given the diagnoses of bipolar disorder, ODD, depression, anxiety, PTSD, and schizophrenia. Regarding pharmacotherapy, she had different trials with olanzapine, doxepin, guanfacine, prozac, and seroquel, but was never fully compliant with medication. In the current hospitalization, she was restarted on olanzapine (10 mg HS) and put on lorazepam (0.50 BID), fluoxetine (20 mg QD), prazosin (2 mg HS), and trazodone (25 mg HS). 

Medical history

Besides her psychiatric diagnosis, the patient had been diagnosed with autosomal dominant choreoathetosis with clinical onset at age 3 and had to undergo physical therapy to learn how to walk. Her biological father and sister had the same diagnosis and reportedly other children from the same biological father also had choreiform movements. This condition is associated with intellectual disability, but this patient’s overall cognitive abilities, measured by the Wechsler Adult Intelligence Scale - Fourth Edition (WAIS-IV), fell in the average range (full scale IQ = 98). Her perceptual reasoning index (PRI = 107), working memory index (WMI = 95), and processing speed index (PSI = 108) also fell in the average range. Her verbal comprehension index (VCI = 85) fell in the low average range. This patient’s presentation involved twitching type movements in both lower extremities and occasionally in her arms and upper body that would happen during the day and continue at night even in her sleep. The movements would worsen when she was tired, sick or when she was using substances or undergoing withdrawal from substances. The patient had trouble with running but could otherwise perform activities with no issues. She mentioned she tried baclofen when she was 16 which helped her improve these symptoms. During observation on the unit, her movements demonstrated multifocal myoclonus which accentuate upon exertion but were not distractible. Additionally, there was postural emergence of tremors on extension of arms. 

During her current hospitalization, neurologists reassessed her presentation, diagnosing her with benign hereditary chorea (BHC). Normally, levodopa is usually the first and best treatment for this disease; however, this medication is contraindicated considering the psychotic episode for which she was admitted, which could have been due to schizophrenia. Another medication considered was tetrabenazine, but since the patient was taking fluoxetine, the combination of both medications could prolong the QTc. At last, the decision to administer tetrabenazine was made, to which she seemed to respond favorably having less choreiform movements with this medication. 

Psychological testing

The patient was referred for psychological assessment to assist with diagnostic clarification, treatment planning, and discharge. She completed a clinical interview, the Minnesota Multiphasic Personality Inventory - 3 (MMPI-3), and the Thematic Apperception Test (TAT; Cards 2, 3 GF, 7 GF, 9 GF, 16). The patient’s MMPI-3 profile showed significant elevations on clinical scales designed to measure unusual perceptions or thoughts associated with thought dysfunction. Individuals with these types of elevations may have unrealistic perceptions of what may be happening around them, may have difficulty knowing what is real or imaged, may experience hallucinations, or may believe that other people can hear their thoughts. The patient also had significant elevations on clinical scales measuring antisocial behaviors and externalizing behaviors. Individuals with these types of elevations report difficulties with people in authority, tend to be interpersonally aggressive, and tend to have difficulties associated with uncontrolled behavior (e.g., substance abuse, poor impulse control, violent and abusive behavior). The patient’s profile also showed elevations on scales measuring level of emotional distress and negative emotional experiences. Further examination of special problems scales showed high levels of behavior-restricting fears, anxiety-related experiences such as reexperiencing trauma, stress, helplessness/hopelessness, and eating concerns. She also endorsed significant suicidal ideation. In contrast to a prior diagnosis of bipolar disorder, her profile showed no elevation on a scale measuring hypomania. The patient’s stories on the TAT tended to exhibit odd, bizarre, and unusual content; the characters felt confused and often questioned whether their experiences were real, whether it was imagined, or a dream. In this brief psychological assessment, the patient reported and displayed evidence of a formal thought disorder as evidenced by vague and impoverished speech; thought process that evidenced disorganization, confusion, and thought blocking; and thought content that included auditory hallucinations and delusions (i.e., believing others could hear her thoughts). Her negative urine drug test screen on admission and the persistence and severity of her psychotic symptoms over a 63-day hospitalization was strongly suggestive of schizophrenia spectrum illness, although due to this patient’s various conditions the other diagnoses considered were: post-traumatic stress disorder; cannabis use disorder in early remission, and bipolar disorder by history. 

Interventions

This patient with psychosis presented with a complex set of concerns as she had a family history of mental illness and substance use, a hereditary neurological illness, trauma experiences, and substance use each of which needed to be considered as the treatment team made diagnostic and treatment decisions. The autosomal dominant choreiform disorder was initially thought to not significantly interfere with her cognitive or psychiatric function which is why medications for this disease did not appear warranted. However, later the patient revealed that she was embarrassed by her involuntary movements and would like treatment for them for which the tetrabenazine was initiated with some positive effect on the movement and no adverse reaction on her mood and psychosis. Since she had a prior diagnosis of unspecified bipolar disorder and was presenting with psychotic features, she was referred to psychological assessment to determine if her symptoms were more indicative of a primary psychotic disorder like schizophrenia or bipolar I with psychotic features, which would present with psychotic symptoms only in the context of mania. However, her testing did not suggest hypomania, instead reflecting significant thought disorder and psychosis. Indeed, the patient displayed evidence of a formal thought disorder including vague and impoverished speech; thought process that evidenced disorganization, confusion, and thought blocking and thought content that included auditory hallucinations and delusions. While substance use may produce psychotic symptoms, her symptoms persisted past acute intoxication in spite of a prolonged period of sobriety during a 63-day hospitalization and treatment with antipsychotics. Furthermore, it was thought that her early childhood trauma experiences may have contributed to her psychiatric decompensation. Based on these factors, the treatment team decided that the primary intervention was to treat psychotic symptoms and achieve psychiatric stabilization with a recommendation for ongoing pharmacotherapy. Secondarily, the patient was provided with psychoeducation about the way in which cannabis use may exacerbate psychotic symptoms, and sobriety was strongly recommended. Finally, once the patient had a period of sustained sobriety, engagement in trauma-informed psychotherapy assisted her in better understanding the impact of trauma on her sense of self, others and the world and promoted psychological healing. 

## Discussion

Psychosis consists of hallucinations, delusions, and delusional misidentification syndromes [6]. It is thought that 1.5%-3.5% of people will meet diagnostic criteria for a psychotic disorder, although a significantly larger, variable number will experience at least one psychotic symptom in their lifetime [7]. Psychosis is the defining feature of schizophrenia spectrum disorder and it is both a contributor to disability and a barrier to productivity and participation [6]. Its severity can be defined by the level, number, and duration of psychotic signs and symptoms. In order to define a more severe psychotic disorder, less severe conditions have to be excluded and a thoughtful search for etiological factors that could explain the condition and provide the opportunity for treatment and prevention, has to be conducted [8]. 

It is known that psychosis occurs in a variety of mental and physical disorders, which poses a challenge to identify what provoked a first episode [9]. The neurodevelopmental hypothesis of schizophrenia proposes that environmental risk factors interact with genetic factors during the formation of the nervous system causing subtle abnormalities that leave the individual vulnerable to psychosis later in life [10]. Likewise, it has been stated that the genotype specifies for whom or under what conditions rearing environment is associated with the outcome schizophrenia-spectrum disorder, supporting the “stress-diathesis model” [11]. Furthermore, it has been showed that disordered parenting plays a crucial role in predicting the child’s psychiatric diagnosis, more so than parental psychiatric diagnosis [12]. Overall, the hypothesis of interaction of genotype and environment is supported, as the genotype appears to be sensitive not only to dysfunction in the family environment but also to protective environmental factors [11]. This patient had a history of physical and sexual abuse in early childhood and her biological mother had diagnosis of bipolar disorder, factitious disorder imposed on another, alcohol use, and unspecified personality disorder. These circumstances were most likely contributors to the development of her schizophrenia spectrum illness.

Factors that appear to predict schizophrenia include problems in motor and neurological development, poor social competence, attention and verbal short-term memory deficits, positive formal thought disorder-like symptoms, higher scores on psychosis-related scales in the MMPI, and severe instability of early rearing environment [13]. Furthermore, factors such as low IQ, poorer educational qualifications, cannabis dependence, alcohol dependence, victimization, stressful life events, and neurotic symptoms have been independently associated with psychotic symptoms [14]. In addition, this patient had a diagnosis of BHC which on its own has been related to anxiety, social embarrassment, school problems, or behavioral difficulties [15]. Depression, apathy, psychosis, obsessive compulsive disorder (OCD), attention deficit hyperactivity disorder (ADHD), and prolonged inpatient psychiatric admission have been also been described in early reports of patients with BHC clinical [16]. 

## Conclusions

In young patients, psychotic illnesses are often misdiagnosed, especially at the initial presentation. This could be due to an overlap in symptoms between different syndromes and confounding risk factors such as traumatic experiences and child maltreatment which certainly presents a challenge in emotionally disturbed youngsters. A clear example is the finding that abuse and neglect are risk factors for the later development of borderline personality disorder, which includes brief psychotic symptoms as part of its diagnostic criteria. Therefore, sorting out these different conditions is important to help ensure accuracy of diagnosis and correct treatment. It would also be important to conduct a review of the presence of psychiatric symptoms in BHC to further analyze the interventions and treatment that can be considered in order to improve these patients’ outcomes and overall lifestyle.

## References

[1] Gaebel W, Zielasek J (2015). Focus on psychosis. Dialog Clin Neurosci.

[2] Barch DM, Bustillo J, Gaebel W (2013). Logic and justification for dimensional assessment of symptoms and related clinical phenomena in psychosis: relevance to DSM-5. Schizophr Res.

[3] Stevens JR, Prince JB, Prager LM, Stern TA (2014). Psychotic disorders in children and adolescents: a primer on contemporary evaluation and management. Prim Care Companion CNS Disord.

[4] Reimherr JP, McClellan JM (2004). Diagnostic challenges in children and adolescents with psychotic disorders. J Clin Psychiatry.

[5] McClellan J, McCurry C (1998). Neurodevelopmental pathways in schizophrenia. Semin Clin Neuropsychiatry.

[6] Arciniegas DB (2015). Psychosis. Continuum (Minneap Minn).

[7] van Os J, Hanssen M, Bijl RV, Vollebergh W (2001). Prevalence of psychotic disorder and community level of psychotic symptoms: an urban-rural comparison. Arch Gen Psychiatry.

[8] Heckers S, Barch DM, Bustillo J (2013). Structure of the psychotic disorders classification in DSM-5. Schizophr Res.

[9] Bromley S, Choi MA, Faruqui S (2015). First episode psychosis: an information guide: a guide for people with psychosis and their families. https://www.camh.ca/-/media/files/guides-and-publications/first-episode-psychosis-guide-en.pdf.

[10] McGrath JJ, Féron FP, Burne TH, Mackay-Sim A, Eyles DW (2003). The neurodevelopmental hypothesis of schizophrenia: a review of recent developments. Ann Med.

[11] Tienari P, Wynne LC, Sorri A (2004). Genotype-environment interaction in schizophrenia-spectrum disorder. Long-term follow-up study of Finnish adoptees. Br J Psychiatry.

[12] Johnson JG, Cohen P, Kasen S, Smailes E, Brook JS (2001). Association of maladaptive parental behavior with psychiatric disorder among parents and their offspring. Arch Gen Psychiatry.

[13] Niemi LT, Suvisaari JM, Tuulio-Henriksson A (2003). Childhood developmental abnormalities in schizophrenia: evidence from high-risk studies. Schizophr Res.

[14] Verdoux H, van Os J (2002). Psychotic symptoms in non-clinical populations and the continuum of psychosis. Schizophr Res.

[15] Bird TD, Carlson CB, Hall JG (1976). Familial essential ("benign") chorea. J Med Genet.

[16] Peall KJ, Kurian MA (2015). Benign hereditary chorea: an update. Tremor Other Hyperkinet Mov (NY).

